# Clinical Effects of Glucagon-Like Peptide-1 Agonist Use for Weight Loss in Women With Polycystic Ovary Syndrome: A Scoping Review

**DOI:** 10.7759/cureus.66691

**Published:** 2024-08-12

**Authors:** Melissa Frangie Machado, Taylor Shunk, Grace Hansen, Charles Harvey, Baylee Fulford, Shane Hauf, Olivia Schuh, Matthew Kaldas, Elena Arcaroli, Justin Ortiz, Joseph De Gaetano

**Affiliations:** 1 Family Medicine, Nova Southeastern University Dr. Kiran C. Patel College of Osteopathic Medicine, Tampa, USA; 2 Family Medicine, Nova Southeastern University, Fort Lauderdale, USA

**Keywords:** weight loss and obesity, resistance to insulin, metabolic disorder, women and health, polycystic ovary syndrome (pcos), glucagon-like peptide-1 receptor agonist

## Abstract

Glucagon-like peptide-1 (GLP-1) is a gastrointestinal regulatory hormone that stimulates insulin release from the pancreas. While GLP-1 receptor agonists (GLP-1 RAs) have traditionally been utilized to address insulin resistance, their potential application in treating polycystic ovary syndrome (PCOS) has recently garnered attention. This study aimed to investigate the therapeutic efficacy of GLP-1 RAs use for weight loss in women diagnosed with PCOS.

We conducted a scoping review following the Joanna Briggs Institute (JBI) methodology and adhering to the Preferred Reporting Items for Systematic Reviews and Meta-Analyses (PRISMA) guidelines. Our investigation delved into the clinical effects experienced by women of diverse racial and ethnic backgrounds with PCOS who were prescribed GLP-1 RAs for weight loss. Peer-reviewed articles from Ovid Medline, Web of Science, CINAHL, Cochrane CENTRAL, SCOPUS, and ClinicalTrials.gov spanning from 2012 to 2023 were scrutinized. After eliminating duplicates, 811 articles were identified, and ultimately, eight met the eligibility criteria for inclusion. All studies were published in English and exhibited wide geographic diversity.

The included studies uniformly reported reductions in weight and body mass index (BMI) among patients who were prescribed GLP-1 RAs, specifically liraglutide or exenatide. Additionally, evidence pointed towards improvements in anthropometric parameters (MF1) (including total body weight, BMI, reduction in waist circumference, and total fat percentage), glucose homeostasis, cardiovascular inflammatory markers (midregional pro-atrial natriuretic peptide (MR-proANP) and mid-regional pro-adrenomedullin (MR-proADM)), rates of pregnancy, and menstrual regulation. However, findings regarding the impact of GLP-1 RAs on lipid profiles were inconsistent. Although some short-term adverse effects were noted, long-term effects of GLP-1 RAs use remain undetermined.

GLP-1 RA use demonstrated promising clinical outcomes for women with PCOS, including reduced BMI, improved metabolic parameters, menstrual regularity, and increased rates of natural pregnancy. While the current evidence is encouraging, further research is warranted to elucidate both short- and long-term adverse effects of GLP-1 RA therapy for PCOS.

## Introduction and background

Polycystic ovary syndrome (PCOS) is one of the most common endocrine disorders of female reproduction, affecting 6-13% of women of childbearing age [1,2]. In the United States, the economic burden of PCOS was estimated at $8 billion annually in 2020, when considering pregnancy-related and long-term morbidities [3]. Common characteristics of this syndrome include infertility, metabolic syndrome, chronic oligomenorrhea, as well as signs and symptoms of hyperandrogenism, including hirsutism, acne, and male-pattern hair loss [4]. Persistent hyperandrogenemia is associated with impairments in the hypothalamic-pituitary-ovary (HPO) axis, leading to luteinizing hormone (LH) hypersecretion, aberrant oocytes, and follicular maturation, contributing to menstrual dysregulation [5]. Although the Rotterdam criteria have been proposed as a tool for diagnosis (presence of two: androgen excess, ovulatory dysfunction, or polycystic ovaries), metabolic rearrangements are an important component of the pathophysiology of PCOS that can provide targetable therapeutic avenues to ameliorate the burden of this disease [6,7].

Insulin has been proposed as a significant factor in the pathophysiology of PCOS given that the effects of this hormone are contributory to steroid metabolism [8]. Actions of insulin are propagated by insulin receptors found in tissues of the HPO axis as well as steroidogenic tissues, including the ovary adrenal cortex [5]. Women with a PCOS diagnosis have significant insulin resistance [9]. Although disordered insulin action is independent of obesity in PCOS, increased body mass index (BMI) exacerbates insulin resistance, leading to excessive androgen production by the liver and adrenal cortex as well as decreased hepatocyte sex-hormone binding globulin (SHBG) production and metabolic dysfunction in peripheral tissues, such as skeletal muscle and adipose tissue [5,10].

It has been shown that 50% of patients with a PCOS diagnosis are overweight and obese [7,11] given that the insulin resistance implicated in the pathophysiology of this syndrome predisposes women to metabolic derangements such as hyperinsulinemia, hyperglycemia, impaired glucose tolerance, and dyslipidemia [12]. Metabolic syndrome is a significant risk factor for the development of chronic systemic diseases that affect almost every organ system, such as type 2 diabetes mellitus (T2DM) and cardiovascular disease (CVD) [12-14]. Although the first-line interventions involve lifestyle measures [6,15] to promote weight loss and improve insulin resistance, the development and clinical use of targetable pharmacological interventions has become of interest to attain optimal therapeutic results, especially for women with obesity and PCOS.

Glucagon-like-peptide-1 (GLP-1) is a regulatory GI incretin hormone that is secreted in response to oral glucose intake and stimulates the release of insulin from pancreatic beta cells [16-18]. Because GLP-1 secretion has an incretin mechanism that is independent of plasma glucose concentration, pharmacologic analogs have been developed to improve glycemic efficiency in individuals who have impaired glucose tolerance and type 2 diabetes (T1DM) [13]. Various studies have shown that GLP-1 receptor agonists (GLP-1 RAs) increase post-prandial insulin release and inhibit glucagon secretion, all without the risk of hypoglycemia [19]. Moreover, it has been reported that the use of GLP-1 RAs resulted in weight loss via the hormone’s effects on appetite reduction, increased satiety, and significant control of plasma lipid profiles [20,21].

The serendipitous weight loss effects of semiglutide have prompted a robust investigation to conclude its significance as a therapeutic for weight management. Currently, semaglutide is used as an adjunct to lifestyle interventions for the treatment of T2DM and for reducing the risk of cardiometabolic events in patients with cardiovascular disease and T2DM [22]. Although common therapeutics used as adjunct therapies for the management of metabolic syndrome in PCOS include metformin, thiazolidinediones, inositols, dipeptidyl peptidase-IV (DPP-IV) inhibitors, and sodium-glucose cotransporter-2 inhibitors (SGLT-2), the potential utility of GLP-1 RA use remains understudied [7]. Given that these therapeutics have demonstrated efficacy in improving hyperlipidemia, promoting weight loss, and reducing glycated hemoglobin levels, the implementation of GLP-1 RA could lead to improved outcomes in the management of PCOS [7].

Despite the growing popularity and promising outcomes of GLP-1 RAs as weight-loss adjuncts, there is a need for an examination of the available evidence to determine the safety and efficacy of these pharmacotherapies in patients with PCOS [23-26]. We conducted a scoping review to provide a summary of the current available evidence regarding the relevance of GLP-1 agonist use for weight loss in patients diagnosed with PCOS. To our knowledge, no scoping reviews have been conducted on this topic.

## Review

Methods

We conducted a scoping review in accordance with the methodology outlined by The Joanna Briggs Institute Scoping Review Methodology (JBI) [27].

Our study focused on evaluating the correlation between GLP-1 utilization in female patients formally diagnosed with PCOS. The study’s search strategy was developed collaboratively by the first (MF), second (TS), and third (GH) authors along with a medical research librarian. Peer-reviewed articles that were published in English between 2012 and September 2023 were selected. Exclusion criteria were applied to studies involving children (≤ 18 years of age) or postmenopausal women over the age of 65. Additionally, papers were excluded if they did not align with the conceptual framework of our study.

To ensure comprehensive coverage, the initial search was intentionally broad, aiming to minimize the risk of overlooking potentially relevant studies. The foundation of the search strategy was established through an analysis of key terms related to PCOS and GLP-1 receptor agonists, extracted from MeSH and relevant articles in Embase. Authors five through nine then translated this base search strategy to four additional databases: Ovid Medline, Web of Science, CINAHL, and ClinicalTrials.gov. Gray literature was not included in the search process. The Embase search strategy can be found in Appendix 1.

Authors one (MF) and two (TS) conducted a screening of titles and abstracts based on our pre-defined criteria. Any discrepancies were addressed by the third (GH) author and, when needed, were further resolved through discussion amongst authors one through three until a consensus was reached. Full-text screening followed the same approach. The primary author (MF) developed a thematic framework charting form to guide the extraction of variables. Authors five through nine were responsible for completing the data extraction of selected studies, which included details such as study design, recruitment/data collection period, country, setting, sample size and characteristics, study objectives, and outcomes. Relevant data from the studies were entered into a Microsoft Excel (Microsoft Corporation, Redmond, Washington, United States) extraction form for further analysis.

A pair of authors (authors five through nine) independently utilized the Joanna Briggs Institute Appraisal Tools [28,29] to critically appraise each selected study based on its specific methodologies and screen for potential risk of bias. The studies were categorized into risk levels: scores below 50% indicating high risk, scores between 50%-70% indicating moderate risk, and scores above 70% indicating low risk (Appendices 2 and 3). 

Data from each cell of our framework underwent analysis to comprehensively describe the studies and findings regarding GLP-1 use among patients with PCOS.

Results

**Figure 1:**
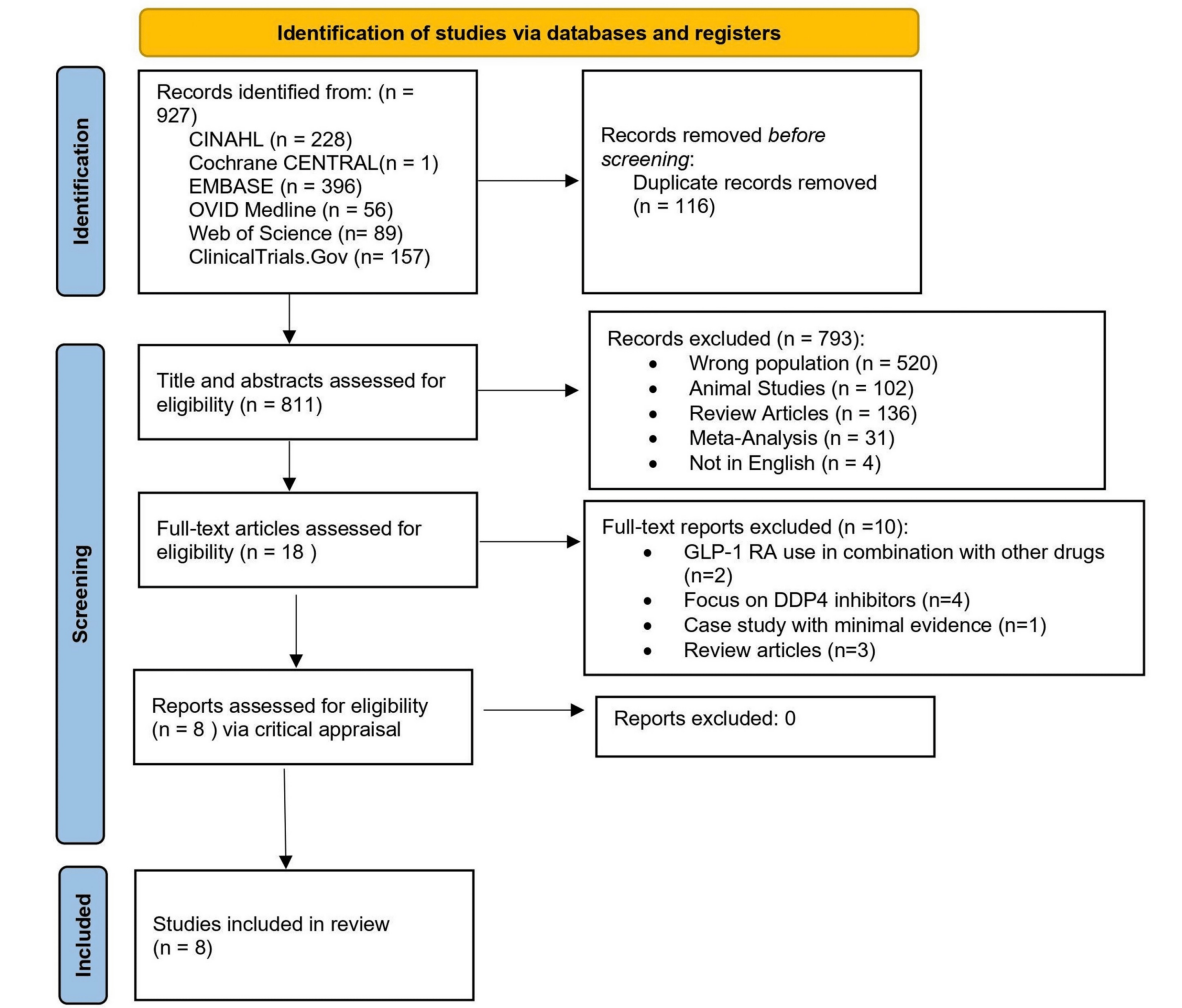
PRISMA flow diagram PRISMA: Preferred Reporting Items for Systematic Reviews and Meta-Analyses

After conducting final searches in Ovid Medline, Web of Science, CINAHL, SCOPUS, and ClinicalTrials.gov, a total of 927 articles were identified. These articles were then imported into EndNote (version 20), where 116 duplicates were identified and removed using the EndNote de-duplication feature. Following de-duplication, the titles and abstracts of 811 articles were evaluated for eligibility, resulting in 18 articles being selected for full-text review. Upon full-text review, eight articles met the inclusion criteria and underwent critical appraisal, with consensus reached among the first three authors [30-37] (Figure 1).

The included studies spanned from 2014 to 2022 [30-37] and exhibited diverse geographical distribution, with representation from various countries including the United States (n = 1) [31], China (n = 2) [33,37], Denmark (n = 3) [34-36], the United Kingdom (n = 1) [30], and the Netherlands (n = 1) [32]. Methodologically, the selected studies included interventional RCT (n = 5) [31-35] and quasi-experimental study methods (n = 3) [30,36,37]. These studies investigated the use of GLP-1 RAs, specifically exenatide (EXE) (n = 3) [30,33,37] and liraglutide (LIRA) (n = 5) [31,32,34-36], in patients diagnosed with PCOS. The average age of study participants ranged from 27.69-35.5 years [30,32-37], while body mass index (BMI) ranged from 28.29 to 40.4 kg/m2 across the studies [30,34,37]. A summary of the characteristics of the included studies is provided in Table 1.

**Table 1:** Summary of included studies RCT: randomized control trial; PCOS: polycystic ovarian syndrome; BMI: body mass index; NIH: National Institutes of Health; lira: liraglutide; PL: placebo; SQ: subcutaneous; EXE: exenatide; TC: total cholesterol; TG: triglycerides; FFA: free fatty acids; LDL-C: low-density lipoprotein cholesterol; HDL-C: high-density lipoprotein cholesterol; NTGC-MS: non-targeted gas chromatography-tandem mass spectrometry; RC: Rotterdam criteria; SHBG: sex hormone binding globulin; FAI: free androgen index; RH-PAT: reactive hyperemia-peripheral arterial tonometry; MR-proADM: midregional-pro-adrenomedullin; MR-proANP: midregional-pro-atrial natriuretic peptide; BID: twice per day; MET: metformin; AMH: anti-Müllerian hormone; VTE: venous thromboembolism; CVD:cardiovascular disease; MRI: magnetic resonance imaging; PAI-1:plasminogen activator inhibitor-1

Citation	Design	Country	Setting	Population	Objective	Methods	Conclusions	GLP1 Regimen
Elkind-Hirsch et al. (2022) [3]	RCT (phase 3)	United States	Outpatient	Nondiabetic, premenopausal women, aged 18–45 years, diagnosed with PCOS (NHI criteria), BMI of at least 30 Kg/m^2^ (N=88)	Investigate the effects of liraglutide (LIRA) on weight, body composition, and hormonal and metabolic parameters.	Study participants were randomly assigned to LIRA (n =55) or PL (n =27) groups.	LIRA 3mg once daily appears superior to placebo in reducing body weight, and androgenicity and improving cardiometabolic parameters in women with PCOS and obesity.	Subcutaneous injection (SQ) of LIRA 3 mg or a visually matching PL once daily for 32 weeks.
Tang et al. (2019) [32]	Clinical interventional trial	China	Outpatient	Overweight/obese women diagnosed with PCOS diagnosed according to the Endocrine Society Clinical Practice Guideline and Asia-Pacific criteria and age-matched controls (N=32)	Study the changes in whole metabolites before and after exenatide treatment (EXE) in overweight/ obese PCOS patients.	Fasting blood samples from 67 participants (32 with PCOS, 35 controls) were collected before and after EXTE assays measured blood lipids, glucose, and insulin, including TC, TG, FFA, LDL-C, and HDL-C. Metabolite changes were detected via NTGC-MS, alongside clinical biochemical observations.	NTGC‐MS‐based metabolic pathway analysis revealed that EXE has a beneficial effect on overweight/obese PCOS patients by regulating metabolic disorders, especially amino acid disorders.	Continual SQ of EXE for three months (initial dose of 5 μg twice a day was increased to 10 μg twice a day after 1 month).
Rasmussen et al. (2014) [31]	Observational	Denmark	Outpatient	Overweight/obese women, diagnosed with PCOS according to Rotterdam criteria (RC), who failed to lose weight with metformin and lifestyle interventions and were starting treatment with LIRA in a private gynecology and fertility clinic (N=84)	Evaluate the effect of the LIRA on weight loss in overweight and obese women with PCOS.	Treatment with LIRA was initiated, and variables such as the date of the first prescription, age, initial body weight, height, weight loss at follow-up, and dose were recorded. The treatment duration extended from the date of the first prescription until either the discontinuation of treatment or the date of the last recorded weight loss for patients still undergoing treatment during the study period 08/2010-01/2012.	Treatment with LIRA in combination with metformin and lifestyle intervention resulted in significant weight loss in overweight and obese women with PCOS.	Dose adjustments were made based on efficacy and tolerability, starting at 0.6 mg daily and increasing to 1.2 mg and later to 1.8 mg as needed.
Dawson et al. (2019) [25]	Open-label CT	United Kingdom	Outpatient	Overweight/obese women, diagnosed with PCOS who met all three RC, excluding other conditions, and were not on medications affecting insulin sensitivity or ovarian function (N=30)	Evaluate the effect of EXE on endothelial function, inflammatory markers and blood clot structure and function in overweight/obese women with PCOS.	Patients underwent a 4-month EXE regimen with adherence tracking, alongside baseline and endpoint clinical and biochemical evaluations encompassing serum testosterone, SHBG, FAI, lipid profile, insulin resistance, and liver function. Endothelial function was gauged via RH-PAT, while clot structure and fibrinolysis were analyzed using turbidity and lysis assays.	EXE caused a 3% reduction in weight, and improved serum markers of endothelial function, inflammation, and clot function reflecting an improvement in cardiovascular risk indices in women with PCOS.	EXE 5 mcg bd for 4 weeks then 10 mcg bd for 12 weeks.
Frossing et al. (2018) [27]	RCT	Netherlands	Outpatient	Women diagnosed with PCOS according to RC. (N=72)	Investigate if treatment with LIRA in women with PCOS reduces levels of the cardiovascular biomarkers MR-proADM, MR-proANP, and copeptin.	Participants were randomized in a 2:1 ratio to receive either LIRA or placebo; for every two participants who received LIRA, one participant received the placebo. Data collection methods included measuring body weight with participants wearing light clothes on a calibrated scale, assessing waist circumference between the lowest rib and the iliac crest, and measuring blood pressure after ten minutes of rest in the sitting position. Blood samples were collected in the morning after a ten-hour overnight fast, and a 75g oral glucose tolerance test was performed with blood samples collected at 0, 30, 60, and 120 minutes. Additionally, biomarkers were analyzed before the randomization key was broken using an automated immunofluorescence assay, and MRI scans were performed to measure volumes of visceral and subcutaneous adipose tissue.	LIRA treatment in women with PCOS reduced levels of cardiovascular risk biomarkers MR-proANP by 25% and MR-proADM by 6% (borderline significance) compared with placebo. The decrease in MR-proANP was independently associated with an increase in the heart rate.	SQ 1.8 mg LIRA once daily for 26 weeks.
Liu et al. (2017) [28]	Open-label CT	China	Outpatient	Women diagnosed with PCOS according to RC	To evaluate the effects of EXE on reproductive and metabolic function in overweight/obese PCOS.	176 were randomized to receive either EXE (n=88) or metformin (MET) (n=88) for the first 12 weeks. Then all patients were treated with MET alone during the second 12 weeks. Metabolic parameters were observed at 0 and 12 weeks, and the rate of pregnancy was tracked during the second 12 weeks.	Short-term EXE was linked to significant weight loss and central adiposity reduction, which may further explain the improvements in insulin resistance, inflammatory markers, and menstrual cycle, which may contribute to increasing pregnancy rates in women with PCOS.	EXE 10 μg BID or (MET) 1000 mg BID for 12 weeks followed by MET alone for an additional 12 weeks.
Nylander et al. (2017) [29]	Double-blind RTC	Denmark	Outpatient	Women diagnosed with PCOS according to RC (N=72)	Investigate the effect of liraglutide on markers of ovarian dysfunction: bleeding ratio, ovarian morphology, levels of AMH and androgens, in women with PCOS.	Participants were randomized 2:1 to receive LIRA (n=42) or placebo (n=21), with data collected including body weight, waist circumference, blood pressure, fasting blood samples, and a glucose tolerance test. Biomarkers (MR-proADM, MR-proANP, and copeptin) were analyzed before unblinding, and MRI scans were conducted to measure adipose tissue volumes. elaborate on total numbers in control vs placebo.	Liraglutide was found to ameliorate ovarian dysfunction in overweight women with PCOS. Improved bleeding regularity, reduced levels of free testosterone and substantial weight loss were observed. Liraglutide could serve as a treatment in overweight women with PCOS.	SQ LIRA 1.8 mg/day, starting at 0.6 mg/day for the first week, increasing to 1.2 mg/day for the second week, and maintaining the 1.8 mg/day dosage thereafter for 26 weeks.
Nylander et al. (2017) [30]	Double-blind RTC	Denmark	Outpatient	Women diagnosed with PCOS according to RC (N=72)	Study the effect of liraglutide intervention on markers of VTE and CVD risk, in PCOS.	Participants were randomized 2:1 to receive LIRA (n=48) or placebo (n=24). Changes in plasminogen activator inhibitor-1 (PAI-1) levels and thrombin generation test parameters, along with anthropometric, metabolic, and endocrine measurements (SHBG, insulin, testosterone) were collected at baseline and follow-up visits.	Liraglutide administration resulted in a significant weight reduction and showed trends towards decreased plasminogen activator inhibitor-1 (PAI-1) levels, suggesting potential benefits on markers of VTE and CVD risk, although differences in thrombin generation were not significant compared to placebo.	SQ LIRA 1.8 mg/day, starting at 0.6 mg/day for the first week, increasing to 1.2 mg/day for the second week, and maintaining the 1.8 mg/day dosage thereafter for 26 weeks.

Across the studies, the use of EXE in women with PCOS consistently resulted in weight and BMI reduction [30,33], with some studies indicating significant improvements compared to alternative treatments [33]. However, the effects on waist circumference and blood pressure varied among studies [30,33,37]. Similarly, all included studies that utilized LIRA as an intervention reported weight loss among PCOS patients [31,32,34-36]. Notably, reductions in various anthropometric measures, such as waist circumference, were observed [30,34]. However, the impact on blood pressure following LIRA administration showed no significant improvement [32]. Furthermore, across the studies, both EXE and LIRA treatments were associated with improvements in menstrual cycle frequency and rates of spontaneous pregnancy [31,33,34].

Several studies evaluated the effect of GLP-1 RA on biochemical parameters that are indicative of metabolic health. Across the studies, both EXE and LIRA therapies were associated with reductions in inflammatory markers, including C-reactive protein (CRP) [30,33]. Additionally, improvements in endothelial function and markers of cardiovascular health, such as reduced ICAM-1, p-selectin, e-selectin, serum triglycerides, and baseline thrombogenic potential, were observed following treatment with these medications [30].

Discrepancies in the therapeutic effects of GLP-1 RA use on lipid profiles and sensitivity were evident across the included studies. While some studies reported no significant impact on total cholesterol, low-density lipoproteins (LDL), and high-density lipoproteins (HDL) levels following EXE and LIRA interventions, triglyceride levels exhibited the most variability among the lipid panel components [30-32,34]. Specifically, certain studies found no change in triglyceride levels after pharmacological interventions, whereas others reported significant improvements following GLP-1 receptor agonist use [31,32,34,37]. Additionally, blood glucose parameters were addressed in several studies. Some studies demonstrated improved glucose tolerance and insulin homeostasis with EXE compared to metformin interventions, along with reductions in fasting glucose and HbA1c levels [33]. Similarly, LIRA therapy resulted in improved mean blood glucose and insulin response following a 32-week intervention [31]. However, there were conflicting findings regarding fasting insulin levels, as some studies reported no significant effect [34].

Across the studies, hormonal profiles were examined in relation to GLP-1 receptor agonist interventions. LIRA treatments were consistently associated with decreased testosterone levels and reductions in sex hormone-binding globulin (SHBG) levels [31,32,34]. However, findings regarding the effects of EXE therapy on testosterone levels were inconsistent, with some studies reporting no significant decrease and others noting an increase in SHBG levels [33]. 

Furthermore, both LIRA and EXE interventions were associated with adverse effects, including nausea, vomiting, hypoglycemia, rash, pruritus, bloating, diarrhea, constipation, and gallstone-related pain [33,34,36].

Discussion

PCOS presents multifaceted challenges to women's health, affecting fertility, metabolic well-being, and cardiovascular risk [38]. Exploring novel pharmacological interventions is paramount in addressing this complex condition. In this scoping review, we examined the potential of GLP-1 RAs in managing PCOS, focusing on clinical, biochemical, and adverse effect profiles. The selected studies provided valuable insights into the use of long-acting GLP-1 RAs, including LIRA and EXE, across diverse geographic locations and study designs [30-37]. The findings of this review underscore the growing interest in repurposing GLP-1 RAs beyond their traditional indication for T2DM to address metabolic and hormonal dysregulation and PCOS.

Our review highlighted the favorable effects of GLP-1 RA interventions on clinical parameters, particularly BMI and weight reduction [31,37]. Anthropometric improvements, such as reduction in waist circumference and total fat percentage, were consistently observed following GLP-1 RA therapy [31,37], indicating potential benefits for cardio-metabolic health in PCOS patients. For instance, Dawson et al. reported a 3% reduction in total body weight among PCOS patients who received EXE therapy [30], while Tang et al. reported similar findings, supporting a significant decrease in body weight, BMI, fat content, and waist and hip circumference following EXE intervention [37].

While our study revealed inconclusive gaps in the impact of GLP-1 RAs on lipid profiles, improvements in glucose homeostasis were evident across most studies [30,33-35]. Liu et al. explored the effects of EXE therapy, highlighting improved glucose tolerance in PCOS. These findings reinforce the therapeutic potential of GLP-1 RAs in addressing metabolic dysregulation among PCOS patients.

Moreover, the utilization of GLP-1 RAs has been associated with enhancements in inflammatory and vascular markers, indicative of improved cardiovascular function [30,33]. Reduced levels of CRP and improved endothelial function suggest potential cardio-protective effects of GLP-1 RAs in PCOS patients [30]. Dawson et al. demonstrated enhanced endothelial function and decreased serum triglycerides following EXE treatment [30], while Frossing et al. observed reductions in cardiovascular risk biomarkers, such as MR-proANP and MR-proADM, following LIRA therapy [32], further bolstering the cardiovascular advantages of GLP-1 RA administration for PCOS.

In addressing the reproductive manifestations of PCOS, GLP-1 RA interventions have displayed encouraging results in promoting menstrual regularity and fertility outcomes [31,33]. Reductions in testosterone levels and an increase in SHBG following GLP-1 therapy indicate potential advantages for restoring hormonal equilibrium among PCOS patients [31-35]. For instance, Liu et al. reported improvements in menstrual cycle frequency and rates of spontaneous pregnancy following EXE treatment [33], while Nylander et al. observed improvements in ovarian dysfunction and enhanced bleeding regularity following LIRA intervention [34,35].

Despite these promising findings, caution is warranted regarding the long-term adverse effects of GLP-1 RAs, including gastrointestinal disturbances and hypoglycemia [33-36]. In clinical practice, gastrointestinal disturbances, including nausea, vomiting, and diarrhea, are the most common side effects and reasons for medication discontinuation [39], with pancreatitis being the most frequently reported adverse reaction associated with LIRA and EXE [40]. While LIRA is FDA-approved for chronic weight management, other GLP-1 RAs, such as EXE, used for this purpose are currently prescribed off-label. Further research is needed to elucidate their safety profile. Comprehensive assessments of the adverse effect profiles of GLP1-RAs, including gastrointestinal disturbances and hypoglycemia, are imperative for informing clinical decision-making and optimizing patient care and PCOS management.

Future research endeavors should focus on conducting larger, placebo-controlled trials to establish the efficacy and safety of GLP-1 RAs in PCOS populations. Longitudinal studies are needed to assess the durability of weight loss and metabolic improvements associated with GLP-1 therapy, as well as their impact on cardiovascular outcomes in the long term.

Furthermore, investigating the mechanistic underpinnings of GLP-1 effects on reproductive hormones and fertility outcomes in PCOS is crucial for understanding their potential role in reproductive medicine. Recent research on second-line anti-diabetic medications, including GLP-1 RAs, suggests no increased risk of major congenital malformations with periconceptional maternal use, yet further evidence is required to confirm their safety throughout pregnancy [41]. Additionally, our findings, which show conflicting results regarding the effects of GLP-1 RAs on lipid profiles, underscore the need for more robust studies to clarify their impact on cardiovascular risk factors in PCOS [31,32,34,37].

Our study has several limitations that warrant acknowledgment. The limited number of studies included in this scoping review may restrict the generalizability of our findings. Additionally, the exclusion of studies involving combination therapies and the non-diabetic PCOS population may have influenced the comprehensiveness of our results. Moreover, the recent FDA approval of some GLP-1 RAs for weight management may have limited the availability of eligible studies within our time frame. Furthermore, our reliance on peer-reviewed original articles may have overlooked relevant gray literature and unpublished data. Despite these limitations, our scoping review provides valuable insight into the current landscape of GLP-1 RA use in PCOS and highlights avenues for future research to address existing knowledge gaps and optimize patient care.

## Conclusions

In conclusion, while GLP-1 RAs hold promise as a therapeutic option for PCOS management, further research is warranted to validate their efficacy, safety, and long-term outcomes in this population. As PCOS continues to pose significant clinical challenges, exploring innovative pharmacological strategies remains crucial in improving outcomes and quality of life for affected individuals.

## Appendices

Appendix 1

Search Strategy

All studies before duplicate removal: 927

All studies after duplicate removal: 811

**Table 2:** EMBASE Search (09/29/2023): 396 results

#1	'body weight loss'/de OR 'metabolically benign obesity'/de OR 'normal weight obesity'/de OR 'obesity'/de
#2	'weight loss':ab,ti,kw OR obesity:ab,ti,kw OR overweight:ab,ti,kw
#3	'ovary polycystic disease'/exp
#4	'cystic ovar*':ab,ti,kw OR 'micropolycystic ovar*':ab,ti,kw OR 'multiple follicle cyst*':ab,ti,kw OR 'polycystic ovar*':ab,ti,kw OR 'stein cohen leventhal':ab,ti,kw OR 'stein leventhal':ab,ti,kw OR 'ovar* polycystic':ab,ti,kw
#5	'glucagon like peptide 1 receptor agonist'/exp
#6	'glucagon like peptide 1 receptor agonist*':ab,ti,kw OR 'glucagon like peptide 1 agonist*':ab,ti,kw OR 'glp 1 agonist*':ab,ti,kw OR 'glp 1 receptor agonist*':ab,ti,kw OR 'glucagon like peptide 1 receptor stimulating agent*':ab,ti,kw OR 'glucagon like peptide 1 stimulating agent*':ab,ti,kw OR 'glp 1 receptor stimulating agent*':ab,ti,kw OR 'glp 1 stimulating agent*':ab,ti,kw

Appendix 2

Joanna Briggs Institute (JBI) Checklist Tools for Use in Systematic Reviews

**Table 3:** Joanna Briggs Institute (JBI) checklist for randomized controlled trials (RCT) critical appraisal tool for use in systematic reviews

Criteria	Elkind et al. [31]	Justification	Frossing et al. [32]	Liu et al. [33]	Justification	Nylander et al. [34]	Nylander et al. [35]	Justification
Internal validity								
Was true randomization used for the assignment of participants to treatment groups?	Yes		Yes	Yes		Yes	Yes	
Was allocation to treatment groups concealed?	Yes		Yes	No	Open-label design	Yes	Yes	
Were treatment groups similar at baseline?	Yes		Yes	Yes		Yes	Yes	
Bias related to administration of intervention/exposure								
Were participants blind to treatment assignment?	No	Participants were not blinded due to the nature of the intervention	Yes	No	Open-label design	Yes	No	Participants were not blinded due to the nature of the study
Were outcomes measured in the same way for treatment groups?	Yes		Yes	Yes		Yes	Yes	
Were outcomes measured reliably?	Yes		Yes	Yes		Yes	Yes	
Bias related to participant retention								
Was follow-up complete and if not, were differences between groups in terms of their follow-up adequately described and analyzed?	Yes		Yes	Not mentioned		Yes	Yes	
Statistical conclusion validity								
Were participants analyzed in the groups to which they were randomized?	Yes		Yes	Yes		Yes	Yes	
Was the trial design appropriate and were any deviations from the standard RCT design (individual randomization, parallel groups) accounted for in the conduct and analysis of the trial?	Yes		Yes	Yes		Yes	Yes	

Appendix 3

 *Joanna Briggs Institute (JBI) Checklist for Quasi-Experimental Studies Critical Appraisal Tool for Use in Systematic Reviews*

**Table 4:** Joanna Briggs Institute (JBI) checklist for quasi-experimental studies critical appraisal tool for use in systematic reviews

Criteria	Dawson et al. [30]	Justification	Rasmussen et al. [36]	Justification	Tang et al. [37]
Internal validity					
Is it clear in the study what is the “cause” and what is the “effect” (i.e. there is no confusion about which variable comes first)?	Yes		Yes		Yes
Was there a control group?	No	Open-label methodology	No	No, all participants received intervention (liraglutide) without a comparative group	Yes
Were participants included in any comparisons similar?	No	However, participants had been formally diagnosed with PCOS according to the Rotterdam criteria and met inclusion criteria to ensure homogeneity.	Yes		Yes
Were the participants included in any comparisons receiving similar treatment/care, other than the exposure or intervention of interest?	Yes		Unclear	All participants were women with PCOS who were overweight or obese, and they received similar treatment protocols (liraglutide, metformin, lifestyle intervention) after failing to lose weight with metformin and lifestyle changes alone	Yes
Were there multiple measurements of the outcome, both pre and post intervention/exposure?	Yes		Yes		Yes
Were the outcomes of participants included in any comparisons measured in the same way?	Yes		Yes		Yes
Were outcomes measured in a reliable way?	Yes		Yes		Yes
Was follow-up complete and if not, were differences between groups in terms of their follow-up adequately described and analyzed?	Yes	Follow-up was not complete for all initially enrolled participants due to a high dropout rate (10 out of 30 patients withdrew). Reasons for dropout included side effects of the medication (nausea, vomiting, dizziness) and one patient becoming pregnant.	Yes	Follow-up was not complete for all participants; some were lost to follow-up or discontinued treatment due to side effects or other reasons.	Yes
Statistical conclusion validity					
Was appropriate statistical analysis used?	Yes		Yes		Yes
